# For Whom the Bell Tolls: Acute Kidney Injury and Electronic Alerts for the Pediatric Nephrologist

**DOI:** 10.3389/fped.2021.628096

**Published:** 2021-04-12

**Authors:** Elizabeth D. Nguyen, Shina Menon

**Affiliations:** Division of Pediatric Nephrology, Department of Pediatrics, Seattle Children's Hospital, University of Washington School of Medicine, Seattle, WA, United States

**Keywords:** acute kidney injury, electronic alerts, clinical decision support system, care bundles, electronic health record

## Abstract

With the advent of the electronic medical record, automated alerts have allowed for improved recognition of patients with acute kidney injury (AKI). Pediatric patients have the opportunity to benefit from such alerts, as those with a diagnosis of AKI are at risk of developing long-term consequences including reduced renal function and hypertension. Despite extensive studies on the implementation of electronic alerts, their overall impact on clinical outcomes have been unclear. Understanding the results of these studies have helped define best practices in developing electronic alerts with the aim of improving their impact on patient care. As electronic alerts for AKI are applied to pediatric patients, identifying their strengths and limitations will allow for continued improvement in its use and efficacy.

## Introduction

Acute kidney injury (AKI) is commonly seen in hospitalized children, particularly those who are critically ill, and/or have underlying medical conditions (1, 2). It is independently associated with prolonged hospital stay and an increased risk of mortality (3). Post-hospital discharge, patients with AKI have higher healthcare utilization and are at risk of developing long-term consequences such as proteinuria, reduced renal function, and hypertension (4). Thus, the ability to detect these episodes of AKI can improve the management of these patients to provide the best care possible. However, AKI is often under-recognized and under-documented (5, 6). With the widespread use of electronic health records (EHR), it has become possible to use a variety of clinical decision support systems (CDSS) to improve patient care (7). These include automated alerts that allow providers to receive real-time notifications when triggered by a particular threshold. AKI is an ideal clinical condition for the use of electronic alerts because it has a consensus definition and it can be diagnosed from data available in EHR (8). In this review, we discuss the best practices for AKI alerts, special considerations when developing such tools for children, and its impact on patient outcomes.

## Best Practices for AKI Alerts

The 15th Acute Dialysis Quality Initiative (ADQI) consensus conference focused on utilizing EHR to predict AKI risk and outcomes (8–11). They recognized AKI alerts as an opportunity to prompt earlier evaluation and intervention, and provided guidelines around the development of electronic alert systems. One of the recommendations of the 15th ADQI conference was to further refine the structure of AKI alerts and to link them to actionable interventions for AKI care. More recently, the 22nd ADQI consensus conference provided quality improvement (QI) initiatives around the identification and care of patients with AKI. They recognized the role of bundled interventions in preventing AKI or reducing the severity in patients at risk for AKI. The key recommendations from these consensus conferences are summarized here, highlighting particular challenges when applying these guidelines to pediatric patients (8, 12).

### Purpose of Alert

Alerts associated with AKI serve different purposes depending on the data used to inform the notification and the criteria used to trigger the alert. In particular, some AKI alerts have been designed to identify patients at risk for AKI while others are designed to detect patients currently with AKI (13–15).

#### Identification of Patients at Risk of AKI

The 22nd ADQI emphasized identifying populations at risk of AKI, which can include a baseline set of risk factors including age, medications, baseline creatinine, and a problem list (12). As most pediatric patients inherently have few of these risk factors, efforts in detecting children at risk for AKI have focused on the initiation of nephrotoxic medications. Nephrotoxic Injury Negated by Just-in-Time Action (NINJA) is an ongoing prospective quality improvement project that works to reduce nephrotoxic medication-associated AKI among non-critically ill hospitalized children (12). It involves systematic EHR screening and a decision support process (trigger report). This trigger report is reviewed by pharmacists who recommend daily serum creatinine monitoring in the exposed patients. Upon implementation, it was successful in reducing the number of AKI days per 100 days by 42% in its first year (13), and has since been shown to maintain a 23.8% decrease in AKI rates when incorporated across nine pediatric institutions (16). Risk prediction models have been specifically designed for pediatric patients which incorporate data beyond nephrotoxic agents. Implementation of an AKI risk prediction tool by Driest el al. resulted in increased screening for AKI *via* measurement of serum creatinine in a pediatric intensive care unit (17).

More recently, artificial intelligence and machine learning methods have made it possible to design algorithms to predict future episodes of AKI. Tomašev et al. developed a model for the prediction of AKI using a dataset of more than 700,000 patients from the United States Department of Veterans Affairs (18). Their model was able to predict 55.8% of all inpatient episodes of AKI and 90.2% of all AKI that required subsequent dialysis up to 48 h before they occurred. Sandokji et al. developed a machine learning algorithm that selected 10 factors to predict AKI within a 48-h window specifically in pediatric patients as well as the neonatal population (19). More data on these predictive algorithms and their effect on patient outcomes will be seen in the coming years (20).

#### Identification of Patients With AKI

Most alerts are designed to trigger based on the definition of AKI by the Kidney Disease: Improving Global Outcomes (KDIGO) criteria (21). AKI is defined in stages of increasing severity by a rising serum creatinine from baseline or declining urine output (UOP). Urine output is a simple and sensitive measure of kidney function (22). In the neonatal population, defining AKI by urine output captures more diagnoses than using serum creatinine alone (23). Inconsistent intake and output documentation in the electronic medical record, however, limits the use of UOP as a trigger for alerts. While serum creatinine is a reasonable biomarker to represent a patient's glomerular filtration rate in most cases, creatinine-based alerts alone can produce a false positive rate as high as 30% (24). The recent 23rd ADQI (25) emphasized the utility of incorporating additional injury biomarkers to these systems to select patients most likely to benefit from AKI-specific interventions, but recognize that many of these biomarkers still require clinical validation before widespread use.

### Components of the Alert

The 15th Acute Dialysis Quality Initiative workgroup summary discusses the characteristics of an optimal alert, considering the technological as well as human factors impacting the implementation and efficacy of an electronic alert (8–10). At minimum, the content of the alert should include patient identification, the data used to trigger the alert, and the stage of AKI if available. The alert should occur as close to the time of AKI onset as possible. Some alerts target the primary contact for the patient directly whereas other alerts are displayed in the EHR for any provider with access. Alerts may be passive with no acknowledgment of receipt, or interruptive where a series of actions are required to dismiss the alert or proceed with additional orders.

### Responses to the Alert

While e-alerts have been shown to improve the recognition of AKI, they have not consistently translated into improved outcomes of patients with AKI (15, 26). It has been proposed that alerts should be accompanied by a bundle of diagnostic and therapeutic interventions to prevent further injury. The 22nd ADQI emphasized that every exposure to risk factors associated with AKI, as well as episodes of AKI itself, should be followed by a kidney health response, also called a care bundle (12). Many of these care bundles incorporate the best practices in response to kidney injury in a simple and easy to remember mnemonic (Table 1). While they vary slightly depending on the context of their use, most include similar themes of fluid and blood pressure management, medication review, and the evaluation of urine (31).

**Table 1:** Care bundles for acute kidney injury.

**Name**	**Contents**
4 Ms (12)	Medication adjustment
	Minimize exposure
	Message care team and patient
	Monitor
ABCDE (27)	Address drugs
	Boost blood pressure
	Calculate fluid balance
	Dip urine
	Exclude obstruction
AEIOU (6)	Assess cause of AKI
	Evaluate drug doses
	Intake and output charting
	Optimize volume status
	Urine dipstick
AUDITS (28)	Assessment
	Urinalysis
	Diagnosis
	Investigations
	Treatment
	Seek advice from nephrologist
KAMPS (12)	Kidney function check
	Advocacy
	Medications
	Pressure
	Sick day protocols
KDIGO care bundle for cardiac surgery patients (29, 30)	Avoidance of nephrotoxic agents
	Withhold ACEi and ARBs
	Close monitoring of SCr and urine output
	Avoidance of hyperglycemia
	Consider alternatives to radio-contrast agents
	Optimization of volume status and hemodynamic parameters
WATCH-ME for patients requiring dialysis (12)	Weight assessment
	Access
	Teaching
	Clearance
	Hypotension
	Medications

## Use of AKI Alerts in Adults

The utilization of AKI alerts and its impact on clinical outcomes has been studied in a number of settings throughout the years. Table 2 provides a summary of key publications studying the implementation of AKI alerts, selected from a literature review using the key words “AKI,” “electronic alert,” and “clinical decision support system.” Early studies looked at the impact on e-alert implementation in the recognition of AKI and were helpful in understanding the epidemiology of AKI within the hospital population (41, 42). A study out of Ghent University Hospital by Colpaert et al. (43) was one of the first to report positive clinical outcomes following an automated AKI alert, reporting a significant increase in fluid intervention and a higher proportion of patients returning to baseline kidney function within 8 h of the alert.

**Table 2:** Studies on acute kidney injury alerts.

**References**	**Design**	***N***	**Setting**	**Baseline creatinine definition**	**E-alert type**	**Intervention**	**Key findings**
**Pediatric**							
Menon et al. (6)	Prospective non-randomized	239 AKI alerts in 225 patients	Inpatients (non-ICU) aged 6 mo to 18 yo at Seattle Children's Hospital	Lowest in 6 months prior to admission or eCCl 120 mL/min/1.73 m^2^	Page to primary provider	AEIOU care bundle	• Increase in AKI documentation, adjustment in medications and fluids • Higher eGFR at discharge and follow-up
Gubb et al. (32)	Prospective	2,472 AKI alerts in 1,719 patients	Inpatients ≥ 25 d-old and <18 yo in Wales	eCCl 120 ml/min/1.73 m^2^ or midpoint normative creatinine value for age and sex	Displayed in EHR alongside lab result	None	• Higher 30-day mortality in HA- AKI vs. CA-AKI • Repeated AKI episodes associated with increased 30-d mortality and residual renal impairment
Holmes et al. (33)	Prospective	1,343 AKI alerts	Inpatients and outpatients aged <18 yo in Wales	eCCl 120 ml/min/1.73 m^2^ or midpoint normative creatinine value for age and sex	Displayed in EHR alongside lab result	None	• Greater number of HA-AKI vs. CA-AKI • Improved rate of renal recovery for hospitalized patients
**Adult**							
Wilson et al. (34)	Multicenter, randomized, double blind	3,059 patients in intervention group, 2,971 in usual care group	Inpatient units of 6 hospitals	Lowest in 7 days prior to admission	Pop-up window on EHR	Link to AKI orderset and option to add AKI to problem list	• Overall no change in progression of AKI/death/dialysis • Better AKI documentation • Increased mortality in non-teaching hospitals
Holmes et al. (35)	Prospective	193,838 AKI alerts in 132,599 patients	Inpatient and outpatients >18 yo in Wales	Lowest in last 7 days (HA-AKI) or last 8–365 days (CA-AKI)	Displayed in EHR alongside lab result	None	• Increase in AKI incidence (particularly community-based AKI) • Earlier AKI detection • Improvement in overall mortality
Selby et al. (36)	Multicenter stepped wedge cluster randomized	10,017 AKI alerts	All hospitalized patients >18 yo in five United Kingdom hospitals	Lowest in last 7 days or median of values in prior 8–365 days	Displayed in EHR and phone call to clinic site for AKI stage 2 and 3	AUDITS care bundle	• Increase in AKI documentation, fluid assessment and adjustment in medications • Decrease in hospital length of stay • No change in 30-d mortality
Park et al. (37)	Prospective	1,739 AKI patients after alert implementation	Non-nephrology inpatients in a tertiary referral hospital in Korea	Lowest within 2 weeks or first measured during hospitalization	Pop-up window on EHR	Automatically generated nephrology consult	• Decrease in overlooked and severe AKI events • Increase in nephrology consultation and AKI recovery • No change in mortality
Meersch et al. (29)	Randomized control trial	138 patients in intervention group, 138 patients in control group	Patients undergoing cardiac surgery with CPB at University of Muenster	None; high risk of AKI defined as TIMP2*IGFBP7 ≥0.3	None	KDIGO care bundle for cardiac surgery patients	• Reduction in AKI incidence first 72 h after surgery • Improved hemodynamics • Reduction in rate of moderate-severe AKI
Al-Jaghbeer et al. (38) and Bataineh et al. (39)	Prospective	346,412 AKI patients after alert implementation	All inpatients admitted to adult hospitals within University of Pittsburgh Medical Center system	Lowest in prior 12 months or back-calculation from normal eCCl	Displayed in EHR	Prompt to consult nephrology or ICU	• Decrease in hospital mortality rate, hospital duration and dialysis use • Decrease in nephrotoxic antibiotic use
Kolhe et al. (40)	Prospective observational	1,291 AKI patients after alert implementation	All inpatients > 18 yo at the Royal Derby Hospital	Lowest in last 7 days or median of values in prior 8–365 days	Interruptive alert on EHR requiring acknowledgment	AUDITS care bundle	• Improved mortality • Less progression of AKI • Lower odds of death at discharge.
Wilson et al. (15)	Single blind parallel group randomized control trial	1,201 patients in AKI alert group and 1,192 patients in usual care group	All inpatients > 18 yo at the University of Pennsylvania hospital	Lowest in prior 7 days	Page or email to primary provider	Link to external website with KDIGO AKI practice guidelines	• No change in dialysis requirement, nephrology consults, hospital length of stay

*AEIOU, assess cause of AKI, evaluate drug doses, intake and output charting, optimize volume status, urine dipstick; AKI, acute kidney injury; AUDITS, assessment, urinalysis, diagnosis, investigations, treatment, seek advice from nephrologist; CA-AKI, community-associated AKI; CPB, cardiopulmonary bypass; eCCl, estimated creatinine clearance; EHR, electronic health records; HA-AKI, hospital-associated AKI; ICU, intensive care unit; KDIGO, Kidney Disease: Improving Global Outcomes*.

The first randomized controlled trial on AKI alerts was conducted by Wilson et al. (15) at the University of Pennsylvania. In this single blind, parallel group trial, 1,201 adult patients were assigned to receive an AKI alert and 1,192 were assigned to the usual care group (21). There was no difference between the alert and usual care group in composite relative maximum change in creatinine, dialysis, and death at 7 days. There was no difference in the number of renal consults, number of nephrotoxic agents prescribed, no change in length of stay, and no change in number of creatinine lab measurements within 7 days (15, 44). Interestingly, a secondary analysis of this clinical trial using uplift modeling identified patients who might benefit most from AKI alerts. These were patients at risk of a more slowly developing AKI, including older patients, women, and those with a lower baseline creatinine (45). While pediatric patients were not included in this study, they certainly fall into this category.

Among the largest studies on AKI alerts has been the work of Holmes et al. and the Welsh AKI Steering Group (25, 26, 31, 41, 42). They employed a national AKI alert in a population of more than 3 million people in the hospital as well as the primary care setting. The Wales Laboratory Information Management System tracks creatinine values on patients in real time and an alert is issued according to the KDIGO AKI criteria. Over the course of 4 years, they found that the majority of adult AKI alerts were community acquired (53.5%) vs. hospital acquired (29.3%) and the rest (17.2%) were undetermined (25). They were also able to provide nationwide characterization of AKI in various clinical settings and report the true incidence of AKI in Wales.

Subsequent studies have looked at the impact of pairing the AKI alert with an actionable intervention on clinical outcomes. In the PrevAKI study, Meersch et al. (29) assessed the efficacy of a care bundle based on KDIGO guidelines to prevent cardiac surgery-associated AKI in high-risk patients. Patients were randomized to receive usual care or the KDIGO bundle, which included guidelines for managing volume status, hemodynamics, nephrotoxic agents, and hyperglycemia prevention (Table 1). They found a significantly reduced rate of AKI within 72 h after surgery, as well as improvement in hemodynamic parameters and severity of AKI. There were no changes to dialysis requirements, hospital length of stay, or adverse events related to the kidney.

Kolhe et al. (28) implemented an interruptive EHR alert at the Royal Derby Hospital which forced the provider to override or acknowledge the AKI care bundle. The care bundle required completion before a new blood test or medication could be ordered. The care bundle was completed in 25% patients within 24 h, and case fatality was higher when the care bundle was not completed. With their alert, they found a significant improvement in mortality, less progression to higher AKI stages, and lower odds of death at discharge (40). This study later expanded to the Tackling AKI study, a large multi-center pragmatic stepped wedge cluster randomized trial (36). The interruptive alert was no longer used; instead an alert with the care bundle was displayed in the EHR and a phone call was made to the clinical site for patients with AKI stages 2 and 3. Across five UK hospitals, results were significant for an increase in documentation of AKI. While there were no changes in 30-day mortality, there were improvements in medication optimization, fluid assessment, hospital length of stay, and quality of care (37). One of the reasons cited for the lackluster performance of clinical decision support systems (CDSS) in AKI is the difficulty in achieving effect sizes. Al-Jaghbeer et al. implemented a CDSS for AKI in a large regional health care system (38). They looked at > 500,000 total patients, 12 months before (*n* = 181,696) and 24 months after (*n* = 346,412) alert implementation. The system alerted clinicians on “possible AKI” based on the KDIGO SCr criteria. It also provided information on the reference creatinine used, stage of AKI, and a prompt to consult nephrology or intensive care. In comparing pre- vs. post-alert implementation, they found that mortality rate decreased from 10.2 to 9.4% after alert implementation, and there was a decrease in length of stay from 7.2 to 6.0 days for patients with AKI. A 2-year follow-up study on an additional 337,433 patients demonstrated sustained decrease in mortality rate and length of stay, as well as a significant decrease in the use of nephrotoxic agents (39).

More recently, Wilson et al. looked at e-alerts for AKI in 6,030 patients in a double blinded, parallel, randomized controlled trial across six centers (34). In the electronic health record alerts for acute kidney injury (ELAIA-1) study, they found that patients randomized to alerts were more likely to receive intravenous fluids, get a urinalysis or repeat SCr measured, and have documentation of AKI. There was however no difference in their primary outcome, which was a composite of AKI progression, receipt of dialysis, or death. Interestingly, there was a heterogeneity of treatment effect across the different hospitals. In the non-teaching hospitals in the study, patients in the alert arm were more likely to have met the primary outcome [relative risk (RR) 1.49, 95%CI 1.12–1.98].

## Pediatric AKI Alert Studies

The studies discussed above focused on the adult population. Only a few studies have looked at implementing AKI alerts in pediatric patients (Table 2). The Welsh AKI group studied AKI alerts in pediatric patients in both the hospital and community setting (33). Over a period of 30 months, they reported a total of 2,087 alerts, corresponding to 1,343 incident episodes of AKI, of which 468 occurred in neonates. Hospital-acquired AKI accounted for 40.1%, community-acquired AKI accounted for 29.4%, and the rest was unclassified. They reported an incidence rate of pediatric AKI at 1.37 cases per 1,000 person-years.

A prospective study at Seattle Children's Hospital by Menon et al. (6) aimed to determine whether an AKI alert paired with a standardized care pathway would improve AKI detection and renal outcomes. This study included 239 unique AKI alerts with most being stage 1 AKI (68.6%) and 47% were defined as hospital-acquired AKI. With the alert intervention, this study found a significant increase in AKI documentation, intake and output charting as well as adjustments to fluid and medications. While there was a trend toward decreases in AKI stage, this finding was not statistically significant. Larger multi-center studies with greater longitude will be necessary to better understand the impact of AKI alerts on pediatric patients.

## Limitations

There are limitations in the implementation of AKI alerts, some of which are unique to pediatric patients. Addressing these alerts in future studies may improve their efficacy and interpretability.

### Accuracy of the Alert

The definition of AKI is highly dependent on the reliability and accuracy of information presented in the EHR. Unfortunately, urine output is not documented frequently or accurately enough to use for AKI alerts and a patient's baseline creatinine often does not exist in the medical record. Studies have used different methods to ascertain the baseline serum creatinine (SCr), including using the admission SCr, a pre-admission outpatient creatinine, or nadir inpatient SCr. There are concerns with all methods. For example, if a patient has community-acquired AKI, the admission SCr is likely to be higher than the patient's true baseline resulting in underdiagnosis of AKI (46). An additional issue in pediatrics is that the baseline kidney function evolves as a child grows. This is particularly challenging in neonates as their creatinine at birth is reflective of their mother's kidney function. Using the KDIGO definitions overestimated neonatal AKI in the study done by Holmes et al. (33), and the authors recommend using a serum creatinine >0.5 mg/dl as a threshold for AKI. While imperfect, the most common solutions to calculating baseline creatinine in pediatrics are to estimate baseline SCr including back-calculation based on eGFR of 120 ml/min per 1.73 m^2^ or use a normative midpoint value for age (33, 47).

### Type of Alert

For an alert to work, it must be noticed. Much research has been done on the balance between intrusive and passive alerts and their relative efficacy (7). Providers are more likely to act on an interrupting alert that forces an action. However, if these intrusive alerts are too frequent or disproportionately associated with false positives, all alerts of the same type are more likely to be dismissed without action (48, 49). Improperly implemented alerts can lead to alert fatigue, which may further affect the efficacy of the alert. When considering how to deliver an alert to maximize patient benefit while also reducing alert fatigue, applying alerts only to patients at high risk who may gain most from intervention would be a potential solution (49). Alerts could also be targeted at providers working directly with the patient in question at the time of potential error, such as when nephrotoxic agents are ordered.

### Interventions Associated With Alert

Care bundles have been recommended and used with e-alerts as an attempt to improve the outcomes associated with AKI (6, 28, 29, 38, 40). Currently, care bundles include general common sense measures such as optimal fluid management, medication review, and urinalyses (Table 1). However, as seen in the ELAIA-1 study (34), care bundles that do not provide patient specific recommendations may not be helpful, and have the potential to cause more harm. Additional research is needed on this aspect of CDSS.

## Conclusions

As a tool that is able to detect patients with AKI, electronic alerts meet the need for identifying patients at high risk for poor outcomes. Criticism of existing studies on AKI alerts note that little impact on overall mortality has been seen with the implementation of alerts. However, a higher level of care is consistently provided to patients after AKI alerts were triggered, particularly when bundled with resources of a care plan. Patients with AKI alerts also benefited from detailed documentation of AKI diagnoses, closer attention to fluid and medication management, and the involvement of nephrology providers. This comprehensive level of care that occurs with an automatic real-time notification has few downsides. For pediatric patients in particular, these simple interventions can be an effective resource to reduce the burden of AKI on our communities and hospitals.

## Author Contributions

EN and SM contributed equally to the conception and design of the manuscript, drafted the article and made critical revisions related to the intellectual content of the manuscript, and approved the final version of the article to be published. All authors contributed to the article and approved the submitted version.

## Conflict of Interest

The authors declare that the research was conducted in the absence of any commercial or financial relationships that could be construed as a potential conflict of interest.
